# Entropy Analysis of Neonatal Electrodermal Activity during the First Three Days after Birth

**DOI:** 10.3390/e24030422

**Published:** 2022-03-17

**Authors:** Zuzana Visnovcova, Marek Kozar, Zuzana Kuderava, Mirko Zibolen, Nikola Ferencova, Ingrid Tonhajzerova

**Affiliations:** 1 Biomedical Centre Martin, Jessenius Faculty of Medicine in Martin, Comenius University in Bratislava, Mala Hora 4D, 036 01 Martin, Slovakia; zuzana.visnovcova@uniba.sk (Z.V.); nikola.ferencova@uniba.sk (N.F.); 2 Neonatal Clinic, Jessenius Faculty of Medicine in Martin, Comenius University in Bratislava, University Hospital Martin, Kollarova 2, 036 59 Martin, Slovakia; marek.kozar@uniba.sk (M.K.); kuderava1@uniba.sk (Z.K.); mirko.zibolen@uniba.sk (M.Z.); 3 Department of Physiology, Jessenius Faculty of Medicine in Martin, Comenius University in Bratislava, Mala Hora 4C, 036 01 Martin, Slovakia

**Keywords:** entropy, electrodermal activity, newborns, sympathetic cholinergic nervous system

## Abstract

The entropy-based parameters determined from the electrodermal activity (EDA) biosignal evaluate the complexity within the activity of the sympathetic cholinergic system. We focused on the evaluation of the complex sympathetic cholinergic regulation by assessing EDA using conventional indices (skin conductance level (SCL), non-specific skin conductance responses, spectral EDA indices), and entropy-based parameters (approximate, sample, fuzzy, permutation, Shannon, and symbolic information entropies) in newborns during the first three days of postnatal life. The studied group consisted of 50 healthy newborns (21 boys, average gestational age: 39.0 ± 0.2 weeks). EDA was recorded continuously from the feet at rest for three periods (the first day—2 h after birth, the second day—24 h after birth, and the third day—72 h after birth). Our results revealed higher SCL, spectral EDA index in a very-low frequency band, approximate, sample, fuzzy, and permutation entropy during the first compared to second and third days, while Shannon and symbolic information entropies were lower during the first day compared to other periods. In conclusion, EDA parameters seem to be sensitive in the detection of the sympathetic regulation changes in early postnatal life and which can represent an important step towards a non-invasive early diagnosis of the pathological states linked to autonomic dysmaturation in newborns.

## 1. Introduction

Electrodermal activity (EDA) represents an important psychophysiological index reflecting the cholinergic innervation of sweat glands linked uniquely to the sympathetic nervous system [1,2]. The highly aroused sympathetic branch of the autonomic nervous system is associated with the increase in the sweat gland activity, resulting in changes of the skin conductance. In this context, non-invasive skin surface measurements (predominantly from palmar or plantar eccrine sweat glands) can reveal important information about the level of the physiological (i.e., sympathetic) as well as psychological (e.g., emotional) arousal. In the term of newborns, the monitoring of the EDA may reflect newborns’ actual state of arousal and/or eccrine non-thermal sweating [3]. Additionally, neonatal age is characterized by rapid developmental changes, including the autonomic nervous system, as the principal regulatory mechanism of adaptability and flexibility of the organism. The period after birth is characterized by the sympathetic overactivity and parasympathetic underactivity, as found in several studies (e.g., [4,5]). The autonomic nervous system function can be non-invasively measured from physiologic signals, including heart rate variability (HRV), as a measure of sympathetic and parasympathetic interplay, and therefore ANS functional maturation [6]. While high-frequency HRV reflects mainly cardiac vagal control, low-frequency HRV is mediated by a combination of sympathetic and parasympathetic inputs and baroreflex-linked changes in heart rate [7]. However, there is still discussion about the precise contribution of the sympathetic nervous system to HRV [8]. From this point of view, the non-invasive monitoring of electrodermal activity represents a promising tool for the evaluation of pure regulatory mechanisms within a comprehensive sympathetic network [9].

From the physical aspect, EDA measures the electrical activity of the skin depending on the alteration of eccrine sweat glands activity [9,10,11]. More specifically, a weak electrical current with a constant electrical voltage spreading through two tactile electrodes placed on the body surface causes a change in the balance between anions and cations in the sweat, leading to measurable spontaneously induced EDA fluctuations [9]. In particular, indices of tonic EDA-skin conductance level (SCL)—i.e., evaluating as a mean of slow EDA oscillations reflecting overall conductance and non-specific skin conductance responses (NS.SCRs) and assessing the spontaneous number of waves without external stimuli—represent the appropriate and commonly used parameter for the sympathetic nervous system activity assessment [10,12,13,14]. However, only several studies have focused on tonic EDA evaluation, with controversial findings in newborns. A pioneering study revealed a decreased resting SCL during the second day compared to the first day of life [15]; other studies reported unchanged SCL [16] or even increasing SCL from the first day to the 10th week of life [17]. Additionally, Hernes et al. [17] found higher NS.SCRs during the third day compared to the first day of postnatal life. Further, the EDA changes were assessed by spectral-domain parameters, which describe the spectral arousal through skin surface [1].

Due to the fact that biological systems exhibit nonlinear properties that are difficult to describe only in a linear model, a nonlinear entropy-based time series analysis has been introduced into the EDA recordings, providing a better overview of the biosignal complexity [18,19]. The entropy-based parameters belong to the time-domain analysis of the biomedical signals [20]. The main advantage of entropy methods in clinical research is fast and simple calculations, invariance, and robustness with respect to nonlinear monotonic transformations [21]. Moreover, the entropy algorithm applied to nonstationary data does not require as much, providing more reliable results from short-term recordings compared to other nonlinear indices, such as fractal dimension, correlation dimension, or the Lyapunov exponent [22,23,24,25].

The studies regarding entropy in newborns are rare. Zhang et al. [26] found a significantly higher sample entropy derived from electroencephalogram during active sleep compared to quiet sleep in full-term neonates (aged from 25 to 60 weeks), and other studies revealed a decreased transfer entropy derived from heart rate and respiration records during active sleep compared to quiet sleep in full-term infants [27,28]. However, the entropy-based analysis of the EDA biosignal has not yet been studied in newborns. Thus, we aimed to evaluate the earliest maturation of the sympathetic regulatory integrity, based on using multiple EDA entropy-based indices (approximate entropy, sample entropy, fuzzy entropy, permutation entropy, Shannon entropy, and symbolic information entropy), and additional EDA indices (SCL, NS.SCRs, spectral domain indices in very-low, low, high one, high two, very-high, sympathetic frequency bands, and the normalized EDA index of the sympathetic nervous system) in full-term healthy newborns during the first three days of their life. To the best of our knowledge, this is the first study using the EDA-linked entropy analysis to detect potential sensitive biomarkers for complex sympathetic cholinergic regulation in the first postnatal days.

## 2. Materials and Methods

### 2.1. Participants

The studied cohort consisted of 50 spontaneously born and full-term healthy newborns (21 boys, average gestational age: 39.00 ± 0.21 weeks, average birth weight: 3362 ± 79 g, Apgar scores equal or better than 8 points at 1st min, and 9 points at 5th min and 10th min). The newborns were recruited from the Neonatal Clinic University Hospital Martin. Every newborn was examined by a specialist in neonatology. Exclusion criteria were the following: preterm delivery, presence of a genetic, neurological disorder, acute respiratory, cardiovascular, and other diseases potentially altering the ANS activity. 

### 2.2. Protocol

**Figure 1:**
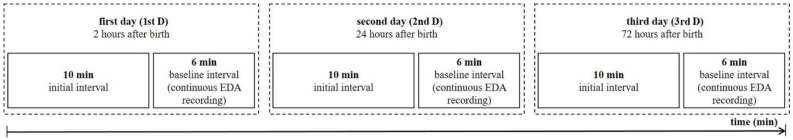
Time schedule of the examination protocol.

The examination was performed in the Neonatal Clinic University Hospital Martin under standard conditions with the minimization of external stimuli (quiet room and stable temperature and humidity). Each newborn was placed in an incubator (1st measurement) or neonatal cot (2nd and 3rd measurements) in a supine position during the examinations. All participants enrolled in this study were in a quiet sleep (closed eyes) with a minimum of body movements occurring during the examination. Newborns in our study were all bathed with water and baby liquid washing product approximately 30 min after birth, because of the possible influence of *vernix caseosa* after birth on the EDA signal; thus, they had clean, dry skin without *vernix* during the examinations. Before the measurement, we used alcohol wipes for carefully cleaning the skin surface of the sole (feet) under the electrodes, according to Boucsein et al. [29]. The sensors (consisting of two dry, Ag-AgCl, bipolar electrodes) for continuous EDA monitoring and recording with a sampling rate of 256 Hz (FlexComp Infinity Biofeedback, Thought Technology, Montreal, QC, Canada) were applied on the dry left sole of the feet according to the recommendation of EDA biosignal measurements [16,30]. The examination protocol consisted of the continual 6 min baseline EDA recording during three periods: 1st day—2 h after birth, 2nd day—24 h after birth and 3rd day—72 h after birth (Figure 1). The initial phase (10 min) was before the baseline phase of study.

### 2.3. Data Analysis

At first before the analysis, the raw EDA records were carefully checked, and the rare artefacts were removed manually for exact data analysis. The tonic component was extracted by the 10th order low-pass finite impulse response filter according to Posada-Quintero et al. [1]. Next, SCL (measured in micro Siemens) was calculated as the average amplitude of the tonic EDA from artefact-free data. For SCL analysis, a 5-min-long EDA signal was used. The index SCL refers to the sympathetic cholinergic nervous system activity [9,31,32]. Further, the other tonic index, NS.SCRs, as an index representing momentary arousal [14], was computed as the frequency of spontaneous skin conductance responses occurring without external stimuli [29].

**Figure 2:**
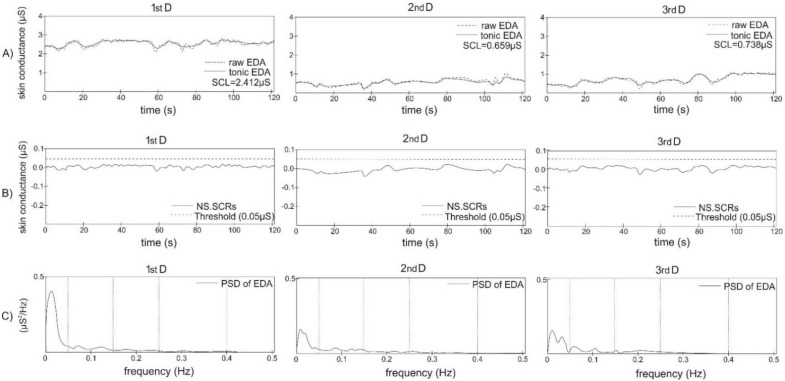
Skin conductance and power spectra of electrodermal activity (EDA) during the 1st day (1st D), 2nd day (2nd D), and 3rd day (3rd D) after birth in selected newborn. (**A**) Raw EDA signal (dashed line), tonic EDA signal (full line), and index skin conductance level (SCL) represents the mean value of tonic EDA (μS); (**B**) non-specific skin conductance responses (NS.SCRs) (number of responses per minute) were obtained by removing the tonic EDA components from the EDA (full line); fixed threshold was used to determine number of NS.SCRs; (**C**) power spectral density (PSD) of EDA (full line);dashed line denotes 0.045 Hz, 0.15 Hz, 0.25 Hz, and 0.40 Hz, respectively.

In a spectral domain analysis, the raw EDA data were filtered with an 8th-order Chebyshev Type I low-pass filter and down-sampled to 2 Hz. Consequently, the data were high-pass filtered with an 8th-order Butterworth filter. The power spectra of EDA signals were evaluated using Welch´s periodogram method with a 50% overlap. The mean power spectrum was calculated by fast Fourier transform (with a Blackman window length of 128 samples) and spectral powers (µS^2^) in the fitting frequency bands (very-low frequency (VLF): 0.000–0.045 Hz; low frequency (LF): 0.045–0.15 Hz; high frequency one (HF1): 0.15–0.25 Hz; high frequency two (HF2): 0.25–0.40 Hz, very-high frequency (VHF): 0.40–0.50 Hz, sympathetic frequency (Symp): 0.045–0.25 Hz, and total) were achieved according to Posada-Quintero et al. [1]. Next, the normalized EDA index of the sympathetic nervous system (EDASymp_n_) (n.u.) was calculated as a ratio between EDASymp and the total power. Spectral-domain indices describe information about the spectral distribution of sympathetic arousal in the skin [1]. Raw EDA, tonic EDA, SCL, NS.SCRs, and power spectra of EDA from the selected newborn during all three days of examination are illustrated in Figure 2.

Finally, the entropy-based parameters (approximate entropy, sample entropy, fuzzy entropy, permutation entropy, Shannon entropy, and symbolic information entropy) were evaluated separately from the individual phases of the protocol using filtered EDA data. 

### 2.4. Approximate Entropy

Approximate entropy (ApEn) informs about the irregularity of the time-series [33]. A lower ApEn is characterized by a high degree of system regularity, and conversely, higher ApEn is characteristic of a more irregular system [34]. ApEn is defined as the probability of similarity between vectors with length, m, and length, m − 1 (from N points long data), within a given tolerance size, r [33].

At first, a sequence of vectors, a(1), a(2), a(3), …, a(N − m + 1) in R^m^, is formed into (N − m + 1) sequences of vectors composed of m consecutive points, as the following:

a(i) = [u(t_i_), u(t_i+1_), u(t_i+2_), …, u(t_i+m−1_)]. (1)

Next, for each i, 1 ≤ i ≤ (N − m + 1), the C_i_^m^ (r) was calculated according to the formula:

(2) $$C_{i}^{m}\left(r\right)=\frac{\mathrm{number}\;\mathrm{of}\;j\;\mathrm{satisfying}\;\mathrm{the}\;\mathrm{following}\;\mathrm{condition}:\;d\left[a\left(i\right),\;a\left(j\right)\right]\leq r}{N-m+1},$$

where $d\left[a\left(i\right),a\left(j\right)\right]$ represents the maximal difference of scalar components of vectors a(i) and a(j), defined as:

(3) $$d\left[a\left(i\right),a\left(j\right)\right]=\max(\;|\;u\left(t_{i+k-1}\right)-u\left(t_{j+k-1}\right)|,\;1\leq k\leq m).$$

Then, Φ^m^(r) was defined as:

(4) $$\Phi^{m}\left(r\right)={\left(N-m+1\right)}^{-1}\;\sum_{i=1}^{N-m+1}\log C_{i}^{m}\left(r\right).$$

Lastly, ApEn is defined as the following:

(5) $$\mathrm{ApEn}\left(m,r,N\right)={\;\Phi }^{m}\left(r\right)-{\;\Phi }^{m+1\;}\left(r\right).$$

The values N (the data length), m (the embedding dimension), and r (the tolerance value) were selected as 300, 2 and 0.2 times the standard deviation of the data, respectively, as they are typically used for clinical data entropy-based analysis [35,36,37].

### 2.5. Sample Entropy

Sample entropy (SampEn) was introduced as an improvement of ApEn, because the calculation of ApEn results in a bias caused by evaluating the self-matching (counts pairs of similar epochs) vectors a(i) in the formula C_i_^m^ (r), which could affect the performance of this statistical measurement [34,38]. To eliminate the self-comparison, the C_i_^m^ (r) is defined as the following:

(6) $$C_{i}^{m}\left(r\right)=\frac{\mathrm{number}\;\mathrm{of}\;j\;\mathrm{satisfying}\;\mathrm{the}\;\mathrm{following}\;\mathrm{condition}:\;d\left[a\left(i\right),\;a\left(j\right)\right]\leq r}{N-m+1},\;(i\;\neq \;j).$$

Then, C^m^(r) is defined as:

(7) $$C^{m}\left(r\right)={\left(N-m+1\right)}^{-1}\;\sum_{i=1}^{N-m+1}C_{i}^{m}\left(r\right).$$

Finally, SampEn is defined as the following:

(8) $$\mathrm{SampEn}\left(m,r,N\right)=\log C^{m}\left(r\right)-\log C^{m+1}\left(r\right).$$

SampEn, similarly to ApEn, describes the irregularity of the clinical and experimental time series [34]. The values N, m, and r were chosen, as in the ApEn algorithm evaluated in this study [37]. Additionally, the differences between SampEn and ApEn are the following: (A) excluding self-pairing (vectors are not compared to themselves) and (B) considering only the first N − m vectors of length m so that, for i ≤ N − m, both C^m^_i_ and C^m+1^_i_ are defined [34,38,39]. 

### 2.6. Fuzzy Entropy

Fuzzy entropy (FuzzyEn) represents a reduction of the SampEn dependence [39]. The basis of the FuzzyEn principle is the concept of fuzzy sets calculated using the exponential functions exp(−D^m^_ij_)^n^/r without a fixed boundary, as the fuzzy function to obtain a fuzzy measurement of the similarity of two vectors based on their shape, while the Heaviside function represents a conventional two-state classifier, where an input pattern is judged on its grouping into a given class according to whether it met certain exact characteristics required by the relationship, as in the ApEn and SampEn algorithms [39]. The family of exponential functions has the following properties: (A) is continuous (similarity is not abrupt) and (B) is convex (self-similarity is the maximal) [39,40]. FuzzyEn describes the general behavior of a time series and contains the vagueness and ambiguity uncertainties of the system [39,41].

First, all vectors are normalized, as follows:

(9) $$u_{0}\left(t_{i}\right)=\frac{1}{m}\sum_{j=0}^{m-1}u\left(t_{i+1}\right).$$

Then, the distance of vectors d[a(i), a(j)] is calculated as:

(10) $$d\left[a\left(i\right),a\left(j\right)\right]=\max\left(\left|\left(u\left(t_{i+k-1}\right)-{\;u}_{0}\left(t_{i}\right)\right)-\left(u\left(t_{j+k-1}\right)-{\;u}_{0}\left(t_{j}\right)\right)\right|,\;1\leq k\leq m\right).$$

The degree of similarity (D_ij_^m^(r)) is defined as:

(11) $$D_{\mathrm{ij}}^{m}\left(r\right)=\exp(-\left(\frac{d\left[a\left(i\right),a\left(j\right)\right]{)}^{n}}{r}\right),\;\left(i\neq j\right).$$

Next, the Φ^m^(r) is defined as:

(12) $$\Phi^{m}\left(r\right)={\left(N-m\right)}^{-1}\sum_{i=1}^{N-m}({\left(N-m-1\right)}^{-1}\sum_{j=1,\;j\neq i}^{N-m}D_{\mathrm{ij}}^{m}\left(r\right).$$

Finally, the FuzzyEn is calculated as:

(13) $$\mathrm{FuzzyEn}\left(m,n,r,N\right)=\log\Phi^{m}\left(r\right)-\log\Phi^{m+1}\left(r\right).$$

The values N, m, and r were chosen the same as in the ApEn algorithm evaluated in this study. FuzzyEn depends on the embedding dimension m, which represents the length of the subseries being compared, and on the other two parameters that are responsible for confirming or denying the similarity between the subseries. Generally speaking, the parameters related to the last aspect play a key role in obtaining a reliable entropy calculation; their role has been investigated for a number of different entropy algorithms [42] and for the analysis of biological signals [43,44].

### 2.7. Permutation Entropy

Permutation entropy (PermEn) calculates the natural complexity of the time series [21]. The PermEn principle is based on evaluating the probability distribution of permu-tation pattern obtained by counting the occurrence of each permutation pattern in all position sequences [21,45].

First, the time series {a(i), i = 1, 2, …, N} is embedded into an m-dimension state space as the following:

A(j) = [a(j), a(j + τ), …, a(j + (m + 1)τ)],(14)

Next, the sequences with m consecutive values were defined and classified into classes according to the character of values (ascending order, descending order, the smallest, or biggest value in the middle). Then, the probability of each class can be marked as:

(15) $$P\left(v\right)=\frac{\mathrm{Num}\left(v\right)}{N-\left(m-1\right)\tau },1\leq v\leq m!,$$

where Num(v) is the count of each class in sequences. 

Finally, the PermEn is calculated as:

(16) $$\mathrm{PermEn}\;\left(m\right)=-\sum_{v=1}^{m!}P\left(v\right)\ln P\left(v\right).$$

The maximal value of PermEn is ln(m!), when P(v) = 1/(m!). The values, N and m, were chosen the same as in ApEn algorithm evaluated in this study, and time delay τ was set to 1. The calculation of PermEn is based on the ordinal mapping of the neighboring values into ordinal patterns (embedding vectors), and therefore the selection of the embedding dimension m becomes crucial for obtaining a reliable estimation of complexity [21,46,47,48]. 

### 2.8. Shannon Entropy

Shannon entropy (ShanEn) informs about the randomness and uncertainty of the time series. ShanEn evaluated the complexity of the time series distribution based on the information theory as the information quantification [49,50,51]. Information quantification is defined as the amount of information transmitted in an event and depends on the probability of the event [50].

At first, the time series of EDA is defined as: A = {A(i), i = 1, 2, …, N}, where N is the data length. Consequently, the A series is coarse-grained by a uniform quantization method with pattern L of delayed samples into

A_L_^ξ^ = (A^ξ^(i), A^ξ^(i − 1), A^ξ^(i − 2), …, A^ξ^(i − L + 1)), (17)

with A_L_^ξ^ = {A_L_^ξ^(i), i = 1, 2, 3, …, N-L + 1} [51]. 

Then, the ShanEn is calculated as the following:

(18) $$\mathrm{ShanEn}\left(L,\;\xi \right)=-\sum_{i=0}^{N-L+1}p\left(A_{L}^{\xi }\left(i\right)\right)\log\left(p\left(A_{L}^{\xi }\left(i\right)\right)\right)\;,$$

where $p\left(A_{L}^{\xi }\left(i\right)\right)$ is the probability of the $A_{L}^{\xi }\left(i\right)$ pattern of the current value, $A_{L}^{\xi }\left(i\right)$. The values N, L, and ξ were set to 300, 3, and 6, respectively, as is commonly used for clinical data [52].

### 2.9. Symbolic Information Entropy

Symbolic information entropy (SIEn) represents a non-negative measurable explanation of the nonlinear time series complexity [19]. Further, SIEn characterizes the average irregularity of each class’s rate. At first, the time series is normalized. After normalization, the symbol sequences consisting of consecutive values are defined and classified into classes according to the type of consecutive symbols (i.e., consecutive symbols are equal or in increasing or decreasing order) [19]. Then, the probability of each class is computed by:

(19) $$p_{i}=\frac{n_{i}}{N-m+1},\;i=1,\;2,\;3,\;\ldots ,\;M.$$

Finally, the SIEn can be defined as the following:

(20) $$\mathrm{SIEn}\left(m\right)=-\sum_{i=1}^{M}p_{i}P_{i},\;P_{i}\;\left\{\begin{array}{cc} \log_{2}(p_{i}),\;\; & p_{i}>0\\ 0, & p_{i}=0 \end{array}\right.,$$

The value N represents data length (N = 300), m is the embedding dimension (m = 2), n_i_ is the count number of i, and M is the number of sequences.

Additionally, the difference between Shannon entropy and symbolic information entropy is in the coarse-graining time-series algorithm, data transformation into symbol sequences, and size of the embedding dimension [19,52]. 

### 2.10. Statistical Analysis

Statistical analysis was performed by jamovi version 1.6.9 (Sydney, Australia). The Shapiro–Wilk normality statistical test with the null hypothesis that the sampling distribution is Gaussian [53] was used for evaluation data distributions (Gaussian/non-Gaussian). Absolute values of spectral EDA indices (VLF-EDA, LF-EDA, HF1-EDA, HF2-EDA, VHF-EDA, EDASymp, and Total-EDA) differed greatly among individuals; therefore, they were logarithmically transformed for the next statistical analysis. All data were normally distributed; thus, the repeated-measures analysis of variance (ANOVA) was used to test the effect of the periods of SCL, NS.SCRs, logarithmic-transformed spectral EDA parameters, EDASymp_n_, and EDA entropy indices. Next, the statistical test of the main effect was followed by post hoc pairwise comparisons between the different periods, which was corrected using the Bonferroni method. The Bonferroni correction was based on a method that assisted in decision making in studies involving repetitive sampling [54]. This method is often used to adjust probability (*p*) values when performing multiple statistical tests in any context [55]. It is a widely used method in various experimental contexts, including: (A) comparing different groups at baseline, (B) studying the relationship between variables, (C) examining more than one endpoint in clinical studies [56], (D) correcting for ‘experimental’ and ‘family’ error rate in multiple comparisons [57], and (E) as a post hoc test after the analysis of variance [58]. In all statistical tests, a value of *p* < 0.05 (two-tailed) was consider statistically significant. SCL and EDA entropy parameters were expressed as mean ± SD.

## 3. Results

**Figure 3:**
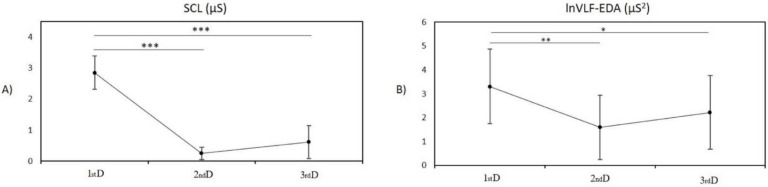
(**A**) Mean ± SD values of skin conductance level during the 1st day (1st D), 2nd day (2nd D), and 3rd day (3rd D) in newborns; (**B**) mean ± SD values of logarithmic-transformed spectral index of EDA in very low frequency band during the 1st day, 2nd day, and 3rd day after birth in newborns. Stars indicate significant differences between periods; * *p* < 0.05, ** *p* < 0.01, and *** *p* < 0.001.

The main effect of periods for the SCL index, evaluated by the repeated-measures ANOVA, was significant (F_[2]_ = 20.20, *p* < 0.001). A pairwise comparison with the Bonferroni adjusted *p* revealed a significantly lower SCL during the second day (*p* < 0.001) and third (*p* < 0.001) day compared to the first day. No significant change in SCL was found between the second and third days (*p* = 0.665, Figure 3A).

The main effect of periods for spectral EDA indices was significant only for lnVLF-EDA (F_[2]_ = 6.47, *p* = 0.004). The pairwise comparison revealed significantly lower lnVLF-EDA during the second day (*p* = 0.007) and third day (*p* = 0.023) compared to the first day after birth. No significant changes in lnVLF-EDA was found between the second and third days after birth (*p* = 0.899, Figure 3B).

The main effect of periods for NS.SCRs, and remaining spectral EDA indices (lnLF-EDA, lnHF1-EDA, lnHF2-EDA, lnVHF-EDA, lnEDASymp, lnTotal-EDA, and EDASymp_n_) was without significant changes (*p* = 0.061, *p* = 0.111, *p* = 0.758, *p* = 0.430, *p* = 0.186, *p* = 0.495, *p* = 0.314, and *p* = 0.109, respectively).

### 3.1. Entropy Analysis

The repeated-measures ANOVA to statistically test the effect of periods revealed significant changes in all entropy-based indices: ApEn–F_[2]_ = 16.00, *p* < 0.001; SampEn–F_[2]_ = 7.73, *p* < 0.001; FuzzyEn–F_[2]_ = 4.30, *p* = 0.018; PermEn–F_[2]_ = 5.09, *p* = 0.008; ShanEn–F_[2]_ = 8.98, *p* < 0.001; and SIEn–F_[2]_ = 5.06, *p* = 0.009.

#### 3.1.1. Post Hoc Pairwise Comparison between Measurement in the First Day and Second Day

**Figure 4:**
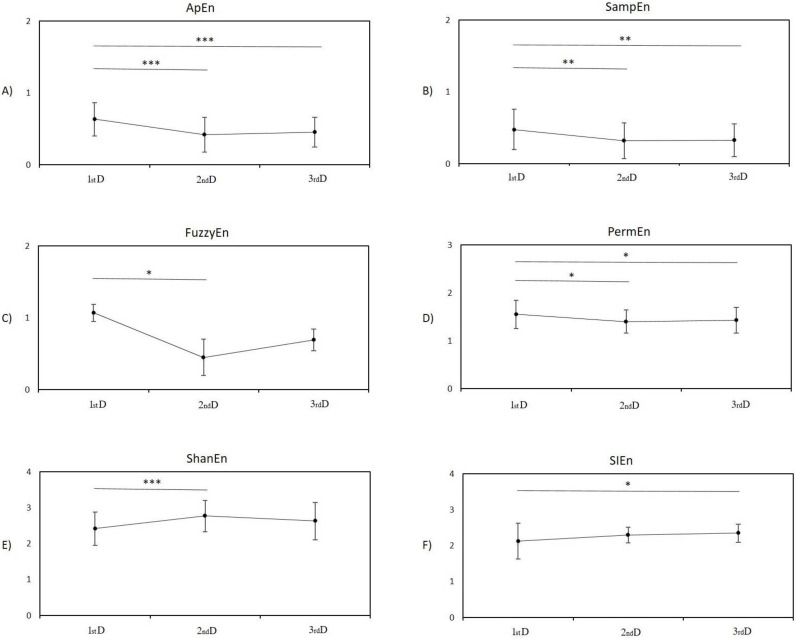
Mean ± SD values of entropy-based indices during the 1st day (1st D), 2nd day (2nd D), and 3rd day (3rd D) after birth in newborns; all evaluated entropy-based indices were significantly affected by the main effect of periods. Post hoc pairwise comparisons between measurement periods were corrected using Bonferroni method. (**A**) Approximate entropy, (**B**) sample entropy, (**C**) fuzzy entropy, (**D**) permutation entropy, (**E**) Shannon entropy, and (**F**) symbolic information entropy. Stars indicate significant differences between periods; * *p* < 0.05, ** *p* < 0.01, and *** *p* < 0.001.

The indices ApEn, SampEn, FuzzyEn, and PermEn were significantly lower during the second day compared to the firstst day (*p* < 0.001 (Figure 4A), *p* = 0.002 (Figure 4B), *p* = 0.019 (Figure 4C), *p* = 0.011 (Figure 4D); respectively). Index ShanEn was significantly higher during the second day compared to the first day (*p* < 0.001, Figure 4E). The index SIEn was without significant changes between these two periods (*p* = 0.065, Figure 4F).

#### 3.1.2. Post Hoc Pairwise Comparison between Measurement in the First Day and Third Day

The indices ApEn, SampEn, and PermEn were significantly lower during the third day compared to the first day (*p* < 0.001 (Figure 4A), *p* = 0.005 (Figure 4B), *p* = 0.049 (Figure 4D); respectively). Index SIEn were significantly higher during the third day compared to the first day (*p* = 0.010, Figure 4F). The indices FuzzyEn, and ShanEn were without significant changes between third and first days (*p* = 0.121 (Figure 4C), *p* = 0.065 (Figure 4E), respectively).

#### 3.1.3. Post Hoc Pairwise Comparison between Measurement on the Second Day and Third Day

No significant changes were found between the second and third days in all entropy-based indices (ApEn: *p* = 0.800 (Figure 4A), SampEn: *p* = 0.950 (Figure 4B), FuzzyEn: *p* = 0.746 (Figure 4C), PermEn: *p* = 0.850 (Figure 4D), ShanEn: *p* = 0.186 (Figure 4E), and SIEn: *p* = 0.770 (Figure 4F)).

## 4. Discussion

The early postnatal age represents an important period characterized by dynamic developmental changes of autonomic, parasympathetic as well as sympathetic, nervous system [59]. A detailed EDA analysis as a marker of the sympathetic nervous system may provide a “window” into the physiological regulatory mechanisms in the earliest postnatal period. In this study, the nonlinear entropy-based EDA analysis revealed several major findings during the first postnatal days (first day—2 h, second day—24 h and third day—72 h afterbirth): (1) the SCL index and spectral parameter lnVLF-EDA were significantly lower during second and third days compared to first day; (2) similarly, the entropy-based indices (ApEn, SampEn, FuzzyEn, and PermEn) significantly decreased during the second and third days compared to the first day; (3) in contrast, the entropy indices ShanEn and SIEn were significantly increased during the second and third days compared to the first day. To the best of our knowledge, this is the first study to reveal the early postnatal dynamic of the sympathetic nervous system using an entropy-based EDA analysis, thus providing novel insights into maturation-linked sympathetic regulatory mechanisms in newborns.

Firstly, SCL represents a traditional index for the evaluation of the eccrine sweat glands activity regulated by postganglionic cholinergic fibers of the sympathetic nervous system [9]. Hernes et al. [17] reported a nonsignificant tendency to decrease in sympathetic activity indexed by lower SCL in the third postnatal day compared to the first day. In our study, the SCL was significantly lower, indicating decreasing sympathetic activity in the third postnatal day compared to the first day after the birth in newborns. Moreover, the spectral index, lnVLF-EDA, was significant decreased, indicating reduced sympathetic cholinergic activity in the second and third postnatal days compared to the first day after the birth in newborns. Further, similarly to SCL and lnVLF-EDA, the entropy indices (ApEn, SampEn, FuzzyEn, and PermEn) assessing the complex properties of the sympathetic cholinergic system showed significantly lower values during the second and third days compared to the first postnatal day. These findings could indicate a decrease in the level of irregularity, or conversely, an increase in the level of regularity [60]. Moreover, these entropy parameters decreasing in the second and third days may be due to the presence of peaks in the signal or combination with the occurrence of peaks and simultaneous rise of regularity [38,60]. In contrast, the indices, ShanEn and SIEn, were higher during the second and third days compared to the first day. This finding, reflecting complex and multicomponent characteristics of the EDA signal, can be explained by mathematical considerations, where ShanEn and SIEn values come from calculating the probability based on the Shannon entropy (i.e., quantify the total amount of information), while the other entropy indices (ApEn, SampEn, and FuzzyEn) use calculations based on embedding, i.e., they calculate the rate of information production [19,21,25,39,60,61]. Moreover, this difference between Shannon and embedding-based entropy indices is according to the study by Li et al. [62], where distribution entropy (as a Shannon-based index) was the opposite compared to FuzzyEn (as an embedding index) derived by the EEG signal in healthy volunteers and patients with epilepsy [62]. However, the calculation of the combination of both entropies based on Shannon as well as the embedding algorithm is recommended for overall signal complex features evaluation [20]. Notably, the embedding indices (ApEn, SampEn, and FuzzyEn) may be more sensitive to detecting changes in EDA dynamics compared to Shannon-based entropies (ShanEn and SIEn) during measurement periods in the crucial neonatal age.

From a physiological aspect, our results could provide novel insights into the developmental regulatory mechanisms of the sympathetic nervous system immediately after the labor and during the earliest postnatal life. More specifically, the labor represents a crucial period, characterized by the transition from internal to external conditions, and causes a stress situation, which is associated with great increasing central sympathetic nervous system outflow to support the fetus during the labor [63]. We suggest that this strong sympathetic activation can be assessed by entropy-based EDA metrics, thus providing important information about labor-induced physiological processes. Further, continual adaptation processes, including central regulatory networks, become active during the first days after the labor. There is an evidence that the areas of the central nervous system, such as the brainstem, reticular formation, premotor cortex, hypothalamus, amygdala, hippocampus, and sympathetic cervical ganglions, are involved in the regulation of the emotional sweating already reflected in EDA changes in the neonatal period [3,64]. In fact, the connectivity between the cortical centers and brain-stem structures regulating the ANS activity increases [59], resulting in the gradual parasympathetic tone raise, as opposed to the sympathetic activity [5]. It seems that ApEn, SampEn, FuzzyEn, and PermEn were the most sensitive to detecting the decreasing sympathetic regulation during the first days of postnatal life in healthy newborns, as found in this study. Therefore, EDA entropy-based measures appear to be sufficiently sensitive to reflect the complexity and nonlinearity of maturation already within the sympathetic regulatory networks during the first postnatal days.

## 5. Conclusions

Our findings revealed that EDA entropy-based measures were sufficiently sensitive to indicate physiological changes of the sympathetic activity in the first postnatal days in healthy and full-term newborns. We assume that a comprehensive EDA analysis could represent an important diagnostic tool for the early detection of pathological states related to sympathetic dysregulation/dysmaturation in the neonatal age.

## Data Availability

Not applicable.
